# Outcome of COVID-19 in patients with idiopathic inflammatory myopathy during the Omicron wave in China: A longitudinal observational study

**DOI:** 10.1371/journal.pone.0317319

**Published:** 2025-02-10

**Authors:** Ying Li, Xiaolan Tian, Chao Sun, Yangyang Wei, Wei Jiang, Linrong He, Chunjia Li, Lu Zhang, Guochun Wang, Xin Lu

**Affiliations:** 1 Peking University China-Japan Friendship School of Clinical Medicine, Beijing, People’s Republic of China; 2 Department of Rheumatology, Key Lab of Myositis, China-Japan Friendship Hospital, Beijing, People’s Republic of China; Kauvery Hospital, INDIA

## Abstract

**Objective:**

The coronavirus disease pandemic brought unknown challenges to patients with idiopathic inflammatory myopathy, who are often heavily immunosuppressed and have comorbidities. We aimed to investigate the outcomes and risk factors of coronavirus disease in Chinese patients with idiopathic inflammatory myopathy during the Omicron wave.

**Methods:**

This observational study included patients with idiopathic inflammatory myopathy who visited the China-Japan Friendship Hospital. Data on baseline characteristics and coronavirus disease-related information were collected through medical records and surveys, and subsequently analysed.

**Results:**

Overall, 204 patients with idiopathic inflammatory myopathy were identified; dermatomyositis was the most common idiopathic inflammatory myopathy subtype. Data were collected from 185 patients with idiopathic inflammatory myopathy who tested positive for severe acute respiratory syndrome coronavirus 2 via polymerase chain reaction or antigen tests; of these, 20 experienced a severe course of the disease, and 9 died. All patients with severe coronavirus disease had idiopathic inflammatory myopathy-associated interstitial lung disease, and the most common antibodies observed in patients with mortality were anti-aminoacyl tRNA synthetase and anti-MDA-5 antibodies. Furthermore, 45.0% of patients in the severe disease group took > 15.0 mg of prednisone daily before infection, a significantly higher proportion than that in the non-severe disease group. Advanced age, mechanics’ hands, dyspnoea, chronic cough and fever during the course of myositis, low lymphocyte count, low serum albumin level, and high D-dimer and ferritin levels before infection were prominent in patients with severe coronavirus disease. Albumin levels below 35.0 g/L and ferritin levels above 306.8 ng/mL were independent risk factors of severe coronavirus disease.

**Conclusion:**

Omicron did not worsen the overall outcomes of coronavirus disease for patients with idiopathic inflammatory myopathy; however, specific risk factors were identified, highlighting the need for targeted management strategies.

## Introduction

In late 2019, a novel coronavirus emerged, leading to a global pandemic. This virus, identified as severe acute respiratory syndrome coronavirus 2 (SARS-CoV-2), is the causative agent of coronavirus disease (COVID-19) [1]. As of 7 January 2024, over 774 million confirmed cases and more than 7 million deaths have been reported globally [2]. Owing to the widespread and continuous evolution of SARS-CoV-2, numerous variants have emerged worldwide during the COVID-19 pandemic period. Omicron subvariants, BA.5.2 and BF.7, with increased transmissibility, extensive immune escape, and a potentially altered host range, became dominant in Beijing since 8 December 2022 [3], bringing a more significant medical burden.

There is a growing body of evidence that SARS-CoV-2 infection is associated with the development and progression of autoimmune diseases. Idiopathic inflammatory myopathy (IIM) is a rare rheumatic disease with a high prevalence of extra-muscular manifestations, particularly interstitial lung disease (ILD). Due to thoracic muscle involvement, ILD, immobility, chronic immunosuppressive treatment, and other comorbidities, SARS-CoV-2 infection with underlying IIM could be a high-risk and life-threatening situation. Considering these factors, it is imperative to explore the risk and outcomes of COVID-19 in patients with IIM. Thus far, some literature has reported the incidence of SARS-CoV-2 infection and the relationship between general factors (such as sex, age, and specific comorbidities) and severe COVID-19 in this population [4–6]. However, no attention has been paid to the significance of baseline characteristics and auxiliary examination results of patients with IIM in predicting adverse outcomes after COVID-19. Specific details regarding the deaths of patients with IIM after SARS-CoV-2 infection are essential to navigate precautions required in future treatment.

On 8 December 2022, SARS-CoV-2 infections were widespread in mainland China due to adjustments in public health control measures. Therefore, we aimed to investigate the outcomes of COVID-19 in patients with IIM and identify the risk factors for severe and critical COVID-19 in this population.

## Materials and methods

This retrospective, longitudinal, observational study involved a cohort of patients diagnosed with IIM at the Department of Rheumatology of China-Japan Friendship Hospital between 8 December 2022 and 5 January 2024.

### Study population

We included 204 patients with IIM who underwent regular follow-ups in our centre before 8 December 2022. The inclusion criteria were a diagnosis of IIM based on the European League Against Rheumatism/American College of Rheumatology classification criteria [7], availability of laboratory auxiliary test results within a month prior to infection, and the ability to understand and complete questionnaires during structured telephone interviews. These interviews were conducted by experienced professionals in our department, who ensured that patients fully understood each question. Patients who lacked baseline data or were unable to cooperate with the investigation were excluded.

Data for this retrospective study were accessed on 8 January 2023 from existing medical records at the China-Japan Friendship Hospital. The study adhered to medical ethical standards and the principles of the Declaration of Helsinki. The Ethical Review Committee of the China-Japan Friendship Hospital approved the study protocol (Approval Number: 2022-KY-156), and it complied with relevant Chinese research regulations. Since we used fully anonymised data from existing medical records without direct human intervention, the need for informed consent was waived by the ethics committee. For the longitudinal observational component involving follow-up surveys, verbal consent was obtained and documented in the medical records of participating patients, following the ethics committee’s guidelines. For minors, consent was obtained from their parents or guardians.

### Clinical data and outcomes of patients collected

Baseline features, including age, sex, clinical manifestations of myositis, details of anti-IIM treatment, complications, and auxiliary examination results within one month before infection, were retrieved from the electronic medical records of each patient in our hospital. Based on our centre’s prior research, parameters such as lymphocyte counts and ferritin levels have demonstrated significant relevance in predicting the prognosis of patients with IIM and in the effective management of complications [8, 9]. Consequently, at each follow-up visit, we collected data on complete blood count, muscle enzyme concentrations, biochemical markers, and immune cell counts to evaluate immune function, monitor treatment efficacy, and identify potential complications.

Data on COVID-19 and vaccination were obtained through the collection of hospital records and/or structured telephone interview questionnaires (S1 Table). The questionnaire included specific questions regarding vaccination dates, types of administered vaccines, and timeline of SARS-CoV-2 infection. To enhance data accuracy, responses were cross-verified with available medical records whenever feasible. To investigate the clinical outcomes of patients with IIM one year after SARS‐CoV‐2 infection, experienced professionals, including physicians and researchers from our department, conducted a one-year follow-up survey on patients who tested positive for SARS-CoV-2 via polymerase chain reaction or antigen tests before 8 January 2023.

Disease progression in patients with IIM was defined as exacerbation of clinical manifestations or an increase in immunosuppressive therapy. According to the United States National Institutes of Health clinical spectrum of SARS-CoV-2 infection [10], COVID-19 severity was categorised as follows: (1) non-severe disease, characterised by the absence of pneumonia or the presence of mild pneumonia and (2) severe disease, marked by symptoms such as dyspnoea, blood oxygen saturation levels below 94% while breathing ambient air, arterial partial pressure of oxygen to fraction of inspired oxygen (PaO2/FiO2) ratio under 300 mmHg, respiratory rate exceeding 30 breaths per min, or lung infiltrates covering more than 50% of the lung field within 24–48 h from symptom onset, as well as the occurrence of organ or multiple organ failure.

### Statistical analyses

Statistical analyses were performed using SPSS software (version 24.0; IBM Corp., Armonk, USA) and GraphPad Prism (8.0.2; GraphPad Software Inc.), and visualised using GraphPad Prism. Categorical variables are expressed as percentages and absolute frequencies. Continuous variables are described using means and standard deviations for normally distributed data and medians with interquartile ranges for non-normally distributed data. Patients were categorised into two groups based on the severity of their COVID-19 infection: severe and non-severe. Group comparisons were conducted using Student’s t-test, Mann–Whitney U-test, chi-squared test, or Fisher’s exact test, as appropriate.

Univariate logistic regression models were used to evaluate the association between identified variables and COVID-19 severity. Variables with a p-value < 0.05 in the univariate binary logistic regression analysis were selected for inclusion in the multivariable binary logistic regression analysis to mitigate excess variables and model instability. This approach aimed to control for confounding effects and identify variables independently associated with COVID-19 severity. A two-sided p-value of < 0.05 was considered statistically significant.

## Results

### Baseline characteristics of patients with IIM

Overall, 204 patients who had been diagnosed with IIM before 8 December 2022 were included. Most (73.0%) patients were female, with a mean age of 51.1 ± 12.7 years. The most common IIM subtype was dermatomyositis (DM) (52.9%), followed by anti-synthetase syndrome (ASS) (27.9%). More than half of the patients had muscle weakness (62.7%), Gottron sign (60.3%), and heliotrope rash (50.4%). ILD was present in 78.9% of patients. Before SARS-Cov-2 infection, more than half of the patients took ≤ 15.0 mg daily dose of prednisone, while 77.9% of patients used one or more immunosuppressants in combination, and 16.7% used Jak inhibitors (Table 1).

**Table 1:** Baseline clinical characteristics of patients with idiopathic inflammatory myopathy.

	Variables	All patients (n = 204)
**General**	Sex (Male, Female)	55:149
	Age (years)	51.1 ± 12.7
	Infected with ^a^SARS-CoV2	185 (90.7)
	Vaccinated against SARS-CoV2	116 (56.9)
**Clinical characteristics**	Duration from the onset of myositis to COVID-19 (months)	22.5 (10.0–46.8)
	Myalgia	93 (45.5)
	Muscle weakness	128 (62.7)
	Dysphagia	35 (17.2)
	Heliotrope rash	103 (50.4)
	Mechanics’ hands	101 (49.5)
	Gottron sign	123 (60.3)
	Dyspnoea	99 (48.5)
	Chronic cough	91 (44.6)
	Fever	58 (28.4)
	Interstitial lung diseases	161 (78.9)
	^*^Other connective tissue diseases	27 (13.2)
	Hypertension	63 (30.8)
	Diabetes mellitus	44 (21.6)
	Heart diseases	36 (17.6)
	Malignancy	8 (3.9)
**treatment**	Prednisone ≤ 15.0 mg/day	114 (55.9)
	Prednisone > 15.0 mg/day	90 (44.1)
	Immunosuppressants	159 (77.9)
	CsA	51 (25.0)
	AZA	12 (5.8)
	MMF	34 (16.7)
	TAC	31 (15.2)
	MTX	29 (14.2)
	CTX	6 (2.9)
	Jak inhibitor	31 (16.7)
	Biologics	5 (2.3)
**Laboratory characteristics**	WBC count (x 10^9^/L)	7.5 (5.4–9.7)
	LYMP count (x 10^9^/L)	1.7 (1.2–2.4)
	CK (IU/L)	56.5 (31.0–150.3)
	LDH (IU/L)	242.5 (201.3–303.0)
	Albumin (g/L)	40.0 ± 4.5
	D-Dimer (mg/L)	0.4 (0.2–0.7)
	IgG (mg/dL)	1170.0 (918.0–1420.0)
	Ferritin (ng/mL)	113.8 (47.3–282.3)
	CD4^+^ T (cell/μL)	704.0 (410.5–1090.3)
	CD8^+^ T (cell/μL)	385.0 (221.5–632.0)
	NK (cell/μL)	155.0 (67.3–259.8)
	CD19^+^ B (cell/μL)	173.0 (90.0–331.5)
**IIM subset**	DM	108 (52.9)
	ASS	57 (27.9)
	IMNM	37 (18.1)
	PM	2 (0.9)

*This category includes a total of 27 cases (13.2% of the total 204), comprising: rheumatoid arthritis (RA) 11 cases (5.4%); systemic lupus erythematosus (SLE) 2 cases (1.0%); spondyloarthritis (SpA) 2 cases (1.0%); Sjögren’s syndrome (SS) 10 cases (4.9%), including both primary and secondary Sjögren’s syndrome; and antiphospholipid syndrome (APS) 2 cases (1.0%).

^a^Abbreviations: SARS-CoV-2, severe acute respiratory syndrome coronavirus 2; CsA, cyclosporine A; AZA, azathioprine; MMF, mycophenolate mofetil; TAC, tacrolimus; MTX, methotrexate; CTX, cyclophosphamide; WBC, white blood cell; LYMP, lymphocytes; CK, creatine kinase; LDH, lactate dehydrogenase; IgG, immunoglobulin G; DM, Dermatomyositis; ASS, Anti-synthetase syndrome; IMNM, Immune-mediated necrotizing myopathy; PM, Polymyositis

As of 8 December 2022, 56.9% of these patients with IIM were completely or incompletely vaccinated against SARS-CoV2. Overall, 185 (90.7%) patients developed SARS-Cov-2 infection-related symptoms before 8 January 2023, tested positive for pathogens, and underwent a one-year follow-up. The other 19 patients, most of whom were completely isolated at home, had no relevant symptoms during this period and tested negative for SARS-Cov-2 (Table 1).

### Incidence and characteristics of severe COVID-19 in patients with IIM

The demographic and clinical characteristics of the 185 IIM with SARS-Cov-2 infection cases in our centre are presented in Table 2. Among them, 20 (10.8%) patients experienced severe disease progression of COVID-19 within one month of infection. No significant difference was observed between the severe COVID-19 and non-severe COVID-19 groups regarding vaccination status, myositis-specific antibodies, comorbidities, and use of immunosuppressive drugs except for prednisone. Of the patients with severe COVID-19, 70% received > 15 mg of prednisone daily before infection, which was a significantly higher proportion than that in the non-severe group (p = 0.008). We also found that patients with a severe disease course were older (p = 0.044) and had a history of mechanics’ hands (p = 0.011), chronic cough (p = 0.003), dyspnoea (p = 0.013), and recurrent fever (p = 0.017) compared with patients with non-severe disease. Notably, all patients with severe COVID-19 had IIM-associated interstitial lung disease (IIM-ILD). Regarding laboratory findings, baseline lymphocyte counts (including CD4+ and CD19+ cells) and albumin levels were lower in patients with severe disease, while D-dimer and ferritin levels were higher.

**Table 2:** Comparison of idiopathic inflammatory myopathy patients with severe COVID-19 and non-severe COVID-19.

Variables	Severe COVID-19 (n = 20)	Non-severe COVID-19 (n = 165)	c^2^/t/Z	p
**Sex, woman (%)**	6 (54.5)	107 (73.3)	1.781	0.182
**Age (years)**	56.7 ± 9.0	50.6 ± 13.0	2.030	0.044
**Duration from the onset of myositis to COVID-19 (months)**	30.5 (8.8–49.3)	23.0 (10.0–48.5)	0.124	0.901
**Vaccine doses (%)**				
**0**	10 (50.0)	70 (42.4)		
**1**	0 (0.0)	3 (1.8)		
**2**	4 (20.0)	28 (17.0)		
**3**	6 (30.0)	64 (38.8)	1.393	0.707
**Myalgia (%)**	11 (55.0)	73 (44.2)	0.833	0.361
**Muscle weakness (%)**	9 (45.0)	109 (66.1)	3.425	0.064
**Dysphagia (%)**	5 (25.0)	28 (17.0)	0.333	0.564
**Heliotrope rash (%)**	9 (45.0)	80 (48.5)	0.087	0.768
**Mechanics’ hands (%)**	15 (75.0)	74 (44.8)	6.496	0.011
**Gottron sign (%)**	13 (65.0)	96 (58.2)	0.343	0.558
**Dyspnoea (%)**	15 (75.0)	75 (45.5)	6.233	0.013
**Chronic cough (%)**	15 (75.0)	66 (40.0)	8.878	0.003
**Fever (%)**	10 (50.0)	41 (24.8)	5.651	0.017
**Interstitial lung diseases (%)**	20 (100.0)	126 (76.4)	4.654	0.031
**Other connective tissue diseases (%)**	5 (25.0)	21 (12.7)	1.324	0.25
**Hypertension (%)**	8 (40.0)	50 (30.3)	0.779	0.394
**Diabetes mellitus (%)**	7 (35.0)	33 (20.0)	1.566	0.211
**Heart diseases (%)**	5 (25.0)	29 (17.6)	0.254	0.614
**Malignancy (%)**	0 (0.0)	8 (4.8)	0.18	0.671
**Prednisone > 15 mg/day (%)**	14 (70.0)	64 (38.8)	7.126	0.008
**Immunosuppressants (%)**	15 (75.0)	128 (77.6)	0.067	0.795
**Jak inhibitor / biologics**	0 (0.0)	31 (18.8)	3.268	0.071
^**a**^ **WBC count (x 10** ^**9**^ **/L)**	6.3 (5.4–8.8)	7.8 (5.5–9.9)	1.808	0.071
**LYMP count (x 10** ^**9**^ **/L)**	1.2 (0.8–1.9)	1.8 (1.3–2.5)	2.520	0.012
**CK (IU/L)**	47.0 (28.0–185.5)	60.0 (33.5–159.5)	0.593	0.553
**LDH (IU/L)**	282.0 (221.8–323.3)	237.0 (201.5–303.0)	1.636	0.102
**Albumin (g/L)**	38.4 (34.3–41.1)	40.5 (37.8–43.4)	2.640	0.008
**D-Dimer (mg/L)**	0.6 (0.3–1.0)	0.4 (0.2–0.7)	2.058	0.040
**IgG (mg/dL)**	1200.0 (1075.0–1615.0)	1190.0 (918.0–1420.0)	1.051	0.293
**Ferritin (ng/mL)**	323.9 (104.0–2131.0)	111.4 (45.1–246.7)	3.233	0.001
**CD4**^**+**^ **T (cell/μL)**	495.5 (316.0–873.5)	793.0 (477.0–1172.0)	2.104	0.035
**CD8**^**+**^ **T (cell/μL)**	316.0 (162.5–551.3)	425.0 (234.0–737.0)	1.624	0.104
**NK (cell/μL)**	104.0 (35.0–261.0)	177.5 (84.5–262.0)	1.577	0.115
**CD19**^**+**^ **B (cell/μL)**	104.5 (66.3–179.0)	200.0 (103.0–374.0)	2.917	0.004
**Anti-MDA5 (%)**	6 (30.0)	54 (32.7)		
**Anti-Mi-2 (%)**	1 (5.0)	4 (2.4)		
**Anti-Jo-1 (%)**	3 (15.0)	14 (8.5)		
**Anti-TIF1γ (%)**	0 (0.0)	8 (4.8)		
**Anti- PL-7 (%)**	3 (15.0)	15 (9.1)		
**Anti-PL-12 (%)**	0 (0.0)	6 (3.6)		
**Anti-EJ (%)**	3 (15.0)	10 (6.1)		
**Anti-NXP2 (%)**	0 (0.0)	4 (2.4)		
**Anti-SAE (%)**	1 (5.0)	1 (0.6)		
**Anti-OJ (%)**	0 (0.0)	1 (0.6)		
**Anti-HMGCR (%)**	0 (0.0)	13 (7.9)		
**Anti- SRP (%)**	1 (5.0)	16 (9.7)		
**MSA negative (%)**	2 (10.0)	19 (11.5)	13.029	0.367
**Death (%)**	9 (45.0)	0 (0.0)	68.627	<0.001

^a^Abbreviations: WBC, white blood cell; LYMP, lymphocytes; CK, creatine kinase; LDH, lactate dehydrogenase; IgG, immunoglobulin G; Anti-MDA5, anti-melanoma differentiation-associated gene 5; Anti-TIF1-γ, anti-transcription intermediary factor 1-gamma; Anti-NXP-2, anti-nuclear matrix protein 2; Anti-SAE, anti-small ubiquitin-like modifier activating enzyme; Anti-HMGCR, anti-3-hydroxy-3-methylglutaryl coenzyme A reductase; Anti-SRP, anti-signal recognition particle; MSA, myositis-specific autoantibody

### Risk factors of severe COVID-19 in patients with IIM

**Fig 1:**
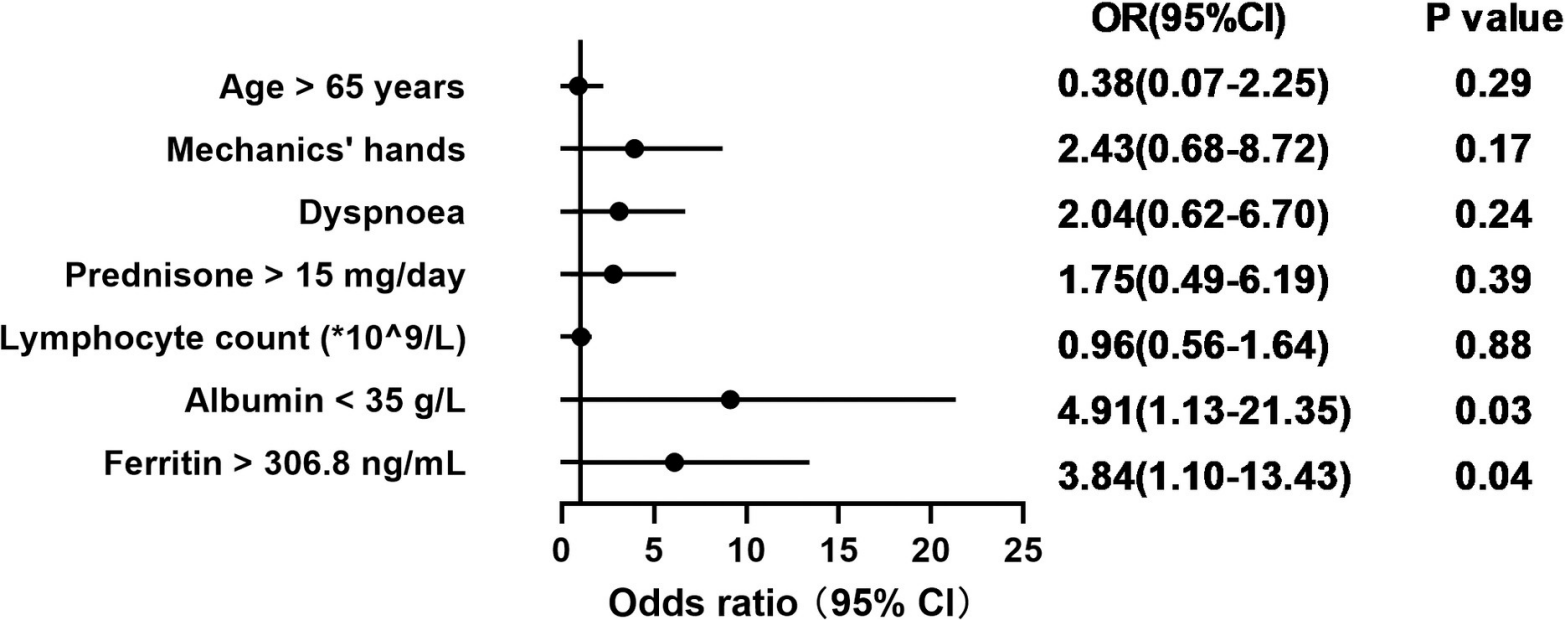
Results of the multivariate logistic regression analysis of risk factors associated with severe COVID-19. Note: Statistics were calculated using binary logistic regression analysis. OR represents the degree of influence of exposure factors. Each risk factor with a p-value < 0.05 in univariate binary logistic regression analysis was entered into the model. Abbreviations: CI, confidence interval; OR, odds ratio.

In the univariate analysis, patients aged over 65 years at the time of COVID-19; those with mechanics’ hands or dyspnoea during the course of myositis; individuals receiving > 15 mg of prednisone daily prior to infection; and those with low lymphocyte counts, albumin levels below 35 g/L, and ferritin levels above 306.8 ng/mL exhibited a higher risk of severe COVID-19. In the multivariate analysis, only albumin levels below 35 g/L (odds ratio [OR]: 4.914, 95% confidence interval [CI]: 1.131–21.346, p = 0.034) and ferritin levels above 306.8 ng/mL (OR: 3.838, 95% CI: 1.096–13.433, p = 0.035) remained independent risk factors of severe COVID-19 (Fig 1).

### Characteristics of patients with IIM who died after infection with COVID-19

Among the cohort of 20 patients diagnosed with severe COVID-19, 9 developed irreversible deterioration, leading to the death of 7 patients within 1 month of infection, while the other 2 died 6 months after the initial infection (Table 3). They all had IIM-ILD; four had the anti-synthetase antibody, three had the anti-MDA5 antibody, one had the anti-signal recognition particle antibody, and one tested negative for the myositis-specific antibody. The main radiographic pattern was non-specific interstitial pneumonia. Pulmonary function testing prior to COVID-19 revealed restrictive ventilatory dysfunction (forced vital capacity < 80% of the predicted value) and moderate diffusion restriction (diffusing lung capacity for carbon monoxide [D_LCO_] between 40% and 60% of the predicted value) in the majority of the 9 patients who died from severe COVID-19. The IIM status of these patients before SARS-Cov-2 infection was mostly active, and they had taken more than 15 mg of prednisone daily (7/9 patients). All these patients died of severe pneumonia. In one case, septic shock was also a factor, while rapid progression of ILD (RP-ILD) might have played a role in another individual’s death.

**Table 3:** Characteristics of patients with idiopathic inflammatory myopathy complicated with severe COVID-19 and poor prognosis.

N	Sex	Age (years)	MSA	CT pattern	Pulmonary Function		IIM stage	Prednisone (mg/day)	Duration from the onset of myositis to COVID-19	Duration from COVID-19 to death	Cause of death
					FVC (%)	D_LCO_ (%)					
1	F	45	MDA5	OP	55.2	49.7	Active	50	26	<1	Severe pneumonia
2	M	43	MDA5	OP	-	-	Active	40	4	<1	Severe pneumonia
3	M	58	PL-7	^a^NSIP	106.4	65.0	Active	27.5	47	<1	Severe pneumonia
4	F	68	Jo-1	NSIP	-	-	Active	30	132	<1	Severe pneumonia
5	F	57	Jo-1	NSIP	50.5	40.7	Active	30	50	<1	Severe pneumonia
6	F	59	Jo-1	NSIP	76.1	45.3	Stable	12.5	13	<1	Severe pneumonia and septic shock
7	M	75	SRP	NSIP	-	-	Active	45	8	<1	Severe pneumonia
8	F	59	MDA5	NSIP	-	-	Active	50	2	6	RP-ILD and severe pneumonia
9	F	47	negative	NSIP	67.9	42.3	Stable	15	38	6	Severe pneumonia

^a^Abbreviations: NSIP, non-specific interstitial pneumonia; MSA: myositis specific antibody; IIM: idiopathic inflammatory myopathy; MDA5, melanoma differentiation-associated gene 5; SRP: signal recognition particle; CT: computed tomography; OP: organising pneumonia; RP-ILD: rapid progression interstitial lung disease; FVC: forced vital capacity; DL_CO_, diffusion capacity of the lungs for carbon monoxide; N, number; F, Female; M, Male

As of 5 January 2024, a total of nine patients had died. Other patients had recovered or improved after treatment, and no new cases of severe COVID-19 were found.

## Discussion

Cases of IIM following hepatitis B, influenza, tetanus, and Bacillus Calmette‒Guérin vaccines have been reported in the literature [11–14]. As the COVID-19 pandemic spread globally, we observed some cases of IIM flare-ups in previously healthy individuals or relapses in patients considered to be in remission or with low disease activity after COVID-19 or SARS-CoV-2 vaccination [15–24], which may be related to the expression of angiotensin-converting enzyme 2, the SARS-CoV2 receptor in skeletal muscle [25]. Notably, MSAs were found in patients vaccinated against SARS-CoV-2 or infected with SARS-CoV-2, especially anti-MDA5 antibodies [15, 26]. These have posed great challenges to patients with IIM and increased the attention directed toward them during the COVID-19 pandemic [27].

Uncertainties on the possibility of an increased risk of COVID-19 due to rheumatic disease still exist, and different studies have reported different conclusions [28–31]. Within one month after the adjustment of the prevention and control policies against COVID-19, the incidence of COVID-19 in patients with IIM amongst our cohort was estimated to be 90.7%, much higher than previous statistics for patients with IIM and the general population [4, 32–35]. This might be related to Omicron, with its high transmission and immune escape ability, as well as the large-scale population movement [3]. The proportion and mortality of severe COVID-19 decreased over time, both in the general population and in individuals with connective tissue diseases (CTDs) [36–38]. In our study cohort, the total proportion of severe COVID-19 cases was 20/185 (10.8%), and the mortality rate was 4.4%, which was similar to or lower than the proportions reported in other CTDs, such as systemic lupus erythematosus, rheumatoid arthritis, and systemic sclerosis [36, 39–41]. The improvement in the outcome may be attributed to the global distribution of the COVID-19 vaccine, antiviral drugs, and SARS-CoV-2 variants with reduced pathogenicity.

Before SARS-Cov-2 infection, certain clinical features and laboratory data of patients with IIM indicated the adverse outcome of COVID-19. We collected the last laboratory data of patients with IIM within one month before infection, which practically reflected their physical state before SARS-CoV-2 infection. We discovered that patients with severe COVID-19 had a lower basal lymphocyte count and albumin level but higher D-dimer and ferritin levels. In addition, albumin and ferritin levels < 35 g/L and > 306.8 ng/mL, respectively, were independent risk factors for severe COVID-19. These clinical features and laboratory values are consistent with the predictors of poor survival in IIM [42–47]. Early identification of these risk factors may enhance monitoring and management of patients with IIM, potentially improving survival rates during pandemics similar to the COVID-19 pandemic.

To date, while some studies suggest that certain disease-modifying antirheumatic drugs (DMARDs), such as rituximab, may increase the risk of severe COVID-19 [48, 49], the overall relationship between DMARDs (including conventional synthetic and biological or targeted synthetic drugs) and severe COVID-19 remains poorly understood. In the current study, we did not find any associations between baseline immunosuppressive drug use and severe diseases, except for the use of glucocorticoids, which was similar to the data reported on 600 patients in the COVID-19 Global Rheumatology Alliance registry [50]. Glucocorticoids are crucial in controlling IIM and preventing disease progression, as well as in managing joint or organ damage associated with persistent inflammation. High-dose glucocorticoid therapy reflects the severity of IIM and is associated with an increased risk of infection secondary to intensified immunosuppressive therapy [47, 51]. The proportion of patients taking more than 15.0 mg of prednisone daily in the severe COVID-19 group was as high as 70%—similar to that in the deceased patients—and was related to IIM activity. This highlights the importance of disease control, preferably by effectively managing DMARD use without increasing glucocorticoid use.

Chronic respiratory diseases may increase SARS-CoV-2 infection or worsen the outcome of COVID-19 [52–57]. ILD is a common extra-muscular manifestation of IIM and is associated with poor prognosis [58–60]. In our study, all patients diagnosed with severe COVID-19 had IIM-ILD, and a high proportion had respiratory symptoms, which may be related to impaired lung reserve and gas exchange, susceptibility to respiratory viruses, and use of corticosteroids and/or immunosuppressants [61–63]. The anti-MDA5 antibody has been found to have a high prevalence and elevated titre in patients with SARS CoV-2 infection, and is associated with higher rates of severe disease [64–66]. However, it is worth noting that most patients who died after infection had ASS as a prototype. Unlike the higher incidence of RP-ILD in anti-MDA5-positive patients, anti-aminoacyl tRNA synthetase positive patients are significantly more likely to develop chronic ILD [67]. Among patients who died during the study period, we found that the disease course of IIM in those with ASS was significantly longer than that of other subgroups, and their lung function test showed restrictive and diffusion reduction patterns. We speculated that the long-term, accumulating inflammatory effect of ILD leads to structural and functional impairment of the lungs, resulting in adverse outcomes. The only death in a patient with immune-mediated necrotizing myopathy (IMNM) was largely attributed to advanced age. The presence of other comorbidities, such as hypertension and diabetes, was not associated with adverse outcomes and is consistent with that observed in the IIM cohorts from the Eastern European region [35].

Infection plays a crucial role in the worsening of interstitial pneumonia [68]. SARS-CoV-2 is thought to destroy the lung tissue by creating an inflammatory response in the form of an immune-mediated injury and direct damage [69, 70]. Two patients died in the sixth month after infection. Although there was no evidence that their deaths were directly related to COVID-19, they all experienced severe COVID-19 in December 2022. The lung function assessment of patient 9 indicated that the D_LCO_ decreased from 3.15 mmol/min/kPa (42.3% of the predicted value) prior to infection to 2.23 mmol/min/kPa (28% of the predicted value) three months post-infection, accompanied by significant progression in imaging. In addition, both patients developed multiple pulmonary infections, including viral, fungal, and bacterial infections; they died of severe pneumonia. Patient 8, who was newly diagnosed with DM before COVID-19 infection, experienced a sharp deterioration in lung function on imaging within one month before her death. We speculated that RP-ILD contributed to the outcome of her death.

Different from previous studies, we analysed the correlation between the clinical characteristics or laboratory data of patients pre-infected with IIM and the prognosis of COVID-19, which makes our conclusions more progressive in guiding clinical diagnosis and treatment. In addition, low serum albumin and high ferritin levels are associated with severe COVID-19 and are also predictors of poor prognosis for IIM, especially IIM-ILD, suggesting that effective clinical management strategies for IIM may provide insights into effective strategies for improving outcomes of severe COVID-19.

Our study has some limitations. First, as IIM is a rare autoimmune disease, the small sample size limited the power of our analyses, particularly for rare events such as death. This also constrained the reliability of our multivariate analyses, as the small number of cases may have reduced the ability to control for confounding factors effectively. In addition, the absence of a population-based comparator made us unable to make comparisons between those with and without COVID-19. Moreover, being a retrospective study, the potential for recall bias exists. However, we sought to minimise this by conducting structured telephone interviews with trained professionals, allowing for real-time clarification of patient responses. Despite these limitations, this study provides important preliminary insights into the clinical outcomes of COVID-19 in IIM patients and highlights risk factors associated with poorer prognoses.

## Conclusions

During the Omicron pandemic wave in China, patients with IIM appeared to have a high incidence of COVID-19 in our centre. However, their clinical outcomes, particularly the rates of severe disease and mortality, did not show significant worsening compared with patients with other CTDs. Risk factors of severe COVID-19 in patients with IIM were low serum albumin and high ferritin levels. Poor outcomes of COVID-19 are more common in patients with ASS who have a long disease course of IIM and poor pulmonary function, followed by patients with anti-MDA5 antibodies. While patients with other subtypes of IIM also contracted COVID-19, the infections did not significantly affect their survival outcomes. Hence, clinicians need to improve the management of patients with ASS and anti-MDA5 positivity during pandemics similar to the COVID-19 pandemics.

## Supporting information

S1 Table Questionnaire on the impact of COVID-19 on patients with idiopathic inflammatory myopathies. (DOCX)
