# Supplementary material for: Outcome of COVID-19 in patients with idiopathic inflammatory myopathy during the Omicron wave in China: A longitudinal observational study
Source: PLoS One. 2025 Feb 10;20(2):e0317319. doi: 10.1371/journal.pone.0317319 (PMC11809795; doi:10.1371/journal.pone.0317319)
Supplement: S1 Table — (DOCX) [file pone.0317319.s001.docx]

**S1 Table. Questionnaire on the Impact of COVID-19 on Patients with Idiopathic Inflammatory Myopathies.**

| **Section 1: Basic Information** | |
| --- | --- |
| **1. Age:** | ○ Under 18 ○ 18–29 ○ 30–39 ○ 40–49 ○ 50–59 ○ 60 and above |
| **2. Gender:** | ○ Male ○ Female |
| **3. Residence:** | ○ City ○ Suburbs ○ Countryside |
| **Section 2: IIM Condition and Medication** | |
| **4. Date of myocarditis onset:** | Year _____ Month _____ Day _____ |
| **5. Type of IIM diagnosed:** | ○ Dermatomyositis ○ Anti-synthetase Syndrome ○ Immune-Mediated Necrotizing Myopathy ○ Other |
| **6. Activity status of IIM before COVID-19 infection:** | ○ Active phase ○ Remission phase |
| **7. Current IIM medications (select all that apply):** | ○ Immunosuppressants ○ Steroids ○ Biologics ○ Antirheumatic drugs ○ Other |
| **8. Steroid details prior to COVID-19 infection:** | Prednisone: _____ mg/day, Dexamethasone: _____ mg/day, Other: _____ mg/day |
| **9. Adjustments to IIM medication during COVID-19 pandemic:** | ○ Yes ○ No |
| **10. Reason for medication adjustment (select all that apply):** | ○ COVID-19 infection ○ Vaccine ○ Supply issues ○ Doctor’s advice ○ Other |
| **Section 3: COVID-19 Vaccination** | |
| **11. COVID-19 vaccine received:** | ○ Yes ○ No |
| **12. Type of COVID-19 vaccine (select all that apply):** | ○ Inactivated ○ mRNA ○ Viral vector ○ Other |
| **13. Number of COVID-19 vaccine doses received:** | ○ 1 dose ○ 2 doses ○ 3 doses ○ 4 doses or more |
| **14. COVID-19 vaccination dates:** | First dose: ____________, Second dose (if applicable): ____________, Booster (if applicable): ___ |
| **15. Interval between vaccine doses:** | (Specify the number of days): ____________ |
| **Section 4: COVID-19 Infection and Hospitalization Details** | |
| **16. Previous COVID-19 infection:** | ○ Yes ○ No |
| **17. Date of COVID-19 diagnosis:** | Year _____ Month _____ Day _____ |
| **18. Method of COVID-19 infection confirmation:** | ○ PCR test ○ Antigen test ○ Antibody test ○ Clinical diagnosis ○ Self-diagnosed ○ Other |
| **19. COVID-19 infection severity:** | ○ Asymptomatic ○ Mild ○ Moderate ○ Severe ○ Critical |
| **20. Symptoms experienced post-COVID-19 infection (select all that apply):** | ○ Asymptomatic ○ Fever ○ Cough ○ Sore throat ○ Shortness of breath ○ Fatigue ○ Muscle/joint pain ○ Loss of taste/smell ○ Chest pain ○ Headache ○ Diarrhea ○ Other |
| **21. Symptom duration:** | ○ <1 week ○ 1–2 weeks ○ 2–4 weeks ○ >4 weeks |
| **22. Hospitalization due to COVID-19:** | ○ Yes ○ No |
| **23. Conditions experienced during hospitalization (select all that apply):** | ○ Severe respiratory symptoms ○ High fever ○ Low oxygen saturation ○ Pneumonia ○ ARDS ○ Sepsis ○ Blood clots ○ Organ failure ○ None ○ Other |
| **24. Lowest oxygen saturation during illness:** | ○ >95% ○ 90–94% ○ 85–89% ○ <85% ○ Not measured |
| **25. Hospital stay duration:** | ○ 1–3 days ○ 4–7 days ○ 8–14 days ○ >14 days |
| **26. Hospitalization reason (select all that apply):** | ○ Severe respiratory symptoms ○ High fever ○ Chest pain ○ Low oxygen levels ○ Dehydration ○ Other |
| **27. Need for oxygen therapy:** | ○ Yes ○ No |
| **28. Type of oxygen therapy used:** | ○ Nasal cannula ○ Face mask ○ High-flow nasal cannula ○ Non-invasive ventilation ○ Invasive |
| **29. ICU admission:** | ○ Yes ○ No |
| **30. ICU stay duration:** | ○ 1–3 days ○ 4–7 days ○ 8–14 days ○ >14 days |
| **31. Medications during hospital stay (select all that apply):** | ○ Antiviral drugs ○ Steroids ○ Antibiotics ○ Monoclonal antibodies ○ Blood thinners ○ Other |
| **32. Complications during hospitalization (select all that apply):** | ○ Pneumonia ○ ARDS ○ Sepsis ○ Blood clots ○ Acute kidney injury ○ Other |
| **Section 5: Additional Information** | |
| **33. Underlying medical conditions (select all that apply):** | ○ Hypertension ○ Diabetes ○ Heart disease ○ Asthma ○ Obesity ○ Other |
| **34. Additional information:** | (Open-ended response) |
